# Impact of Financial Toxicity on the Health‐Related Quality of Life and Financial Well‐Being of Cancer Patients and Survivors: A Comparative Study of the United Kingdom and United States

**DOI:** 10.1002/cam4.70606

**Published:** 2025-01-15

**Authors:** Tran Thu Ngan, Emily Tonorezos, Michael Donnelly, Ciaran O'Neill

**Affiliations:** ^1^ Centre for Public Health Queen's University Belfast Belfast UK; ^2^ Office of Cancer Survivorship, Division of Cancer Control and Population Sciences National Cancer Institute Rockville Maryland USA

**Keywords:** cancer, cancer survivorship, EQ‐5D‐5L, financial toxicity, health‐related quality of life

## Abstract

**Background:**

This study investigated and compared the impact of financial toxicity (FT) on the health‐related quality of life (HRQoL) and financial well‐being of cancer patients and survivors in the United Kingdom (UK) and United States (US).

**Methods:**

UK & US participants (*n* = 600) completed an online questionnaire that consisted of a validated FT instrument (COmprehensive Score for financial Toxicity‐COST), a standardised HRQoL instrument (EQ‐5D‐5L) and questions related to their financial well‐being. Tobit regression models and descriptive statistics plus *χ* ^2^ tests were used to analyse the association between FT and (i) HRQoL whilst controlling for sociodemographic characteristics; and (ii) financial well‐being.

**Results:**

In the UK, health utilities of participants with no assessed experience of FT, mild FT, and moderate/severe FT were 0.81, 0.66, and 0.41, respectively, compared to 0.88, 0.71, and 0.53 in the US. Among those with moderate/severe FT, US participants had significantly higher health utilities compared to their peers in the UK (Mann Whitney test, *p* = 0.0369). In a pooled analysis of UK and US and after controlling for sociodemographic and clinical characteristics, mild and moderate/severe FT was negatively associated with health utilities (*β* coff = −0.13, 95% CI: −0.18, −0.08 and *β* coff = −0.28, 95% CI: −0.34, −0.21, respectively). Over half (54%) of US participants with FT were in debt with median (IQR) debt at I$11,500 (23,000), compared to 32% in the UK with median (IQR) debt at I$ 7200 (12,960). US participants with FT were 2.48 times more likely to be in debt than UK participants with FT (OR = 2.48, 95% CI: 1.46–4.21).

**Conclusions:**

FT is associated with poorer financial well‐being and HRQoL among cancer patients/survivors in the US and UK. The impact of FT on financial well‐being is larger in the US while the impact on HRQoL is worse in the UK. Further studies using prospective data are required to investigate the nature and extent of these relationships.

## 1 Introduction

Financial toxicity (FT) refers to the objective financial burden and subjective financial distress that occurs as a result of cancer diagnosis and treatment [1, 2]. While faced with FT, cancer patients/survivors may experience lower health‐related quality of life (HRQoL) and worse financial well‐being [3, 4, 5, 6, 7]. FT appears to exist in high‐income countries and low‐ and middle‐income countries; and in publicly funded healthcare systems with universal coverage like the United Kingdom (UK) or mixed systems (public and private financed) like the United States (US) [3, 4, 8, 9, 10, 11]. Therefore, investigating FT and comparing the impact of FT in the UK and US with their respectively different health financing mechanisms may afford valuable insights into how FT affects different groups of cancer patients/survivors across health systems with distinct funding arrangements.

This study is the first study in the UK to investigate the impact of FT (using the recognised and accepted definition of FT) on HRQoL [12] though there are several studies in the US that have investigated the topic. Regarding the impact of FT on financial well‐being, US patients and caregivers spent on average $300 out‐of‐pocket (OOP) per month (range: $180–$2598) for cancer‐related expenses in 2018 [13]. A longitudinal study of a nationally representative sample reported that 42.4% cancer patients had depleted their entire life assets within 2 years of diagnosis [14]. In the UK, reports by Macmillan Cancer Support (2013 and 2017) indicated that 83% of cancer patients incurred an average cost of £570 per month as a result of their illness; 80% were financially impacted following their diagnosis; and 40% used savings, sold assets, or borrowed to cover costs related to treatment [15, 16]. However, all these studies used different designs and instruments to measure FT, HRQol, and financial well‐being. These differences make it difficult to undertake a direct comparison of results between countries.

Therefore, this study investigated the impact of FT on financial well‐being and the HRQoL of cancer patients/survivors in the UK and US. Specifically, it investigated the association between FT and financial well‐being and HRQoL, differences between the two countries, and the other factors associated with HRQoL.

## 2 Methods

### 2.1 Study Design and Participants

Data reported in this paper came from a larger 2023 study that investigated FT and its related factors among cancer patients and survivors in the UK & US. The study recruited and collected data through the Prolific research platform (https://www.prolific.co/) (response rate was 94%) using an online structured questionnaire. The questionnaire consisted of the validated FT instrument (COmprehensive Score for financial Toxicity‐COST), standardised HRQoL instrument (EQ‐5D‐5L), and bespoke questions related to participants' financial well‐being.

Inclusion criteria for participants were (i) have or have had cancer (self‐reported when they registered with Prolific before study inception and without knowledge about study inclusion criteria), (ii) aged 18+ years, and (iii) residing in the UK or US. After setting up the inclusion criteria in the platform, an invitation was sent to every eligible individual within Prolific's pool of participants. The platform enabled eligible participants to access the online questionnaire on a first come first serve basis and stopped the access automatically when the predetermined sample size of 600 was reached. More details about study design and methodology are available elsewhere (preprint) [17].

The study was carried out in accordance with the ethical standards of the Helsinki Declaration. Ethical approval No. MHLS 22_178 dated 18th January 2023 was granted by the Faculty of Medicine, Health and Life Sciences Research Ethics Committee, Queen's University Belfast, United Kingdom. The first page of the online survey questionnaire provided information about the purpose of the study, that participation was voluntary, how the study would be conducted, risks and benefits, confidentiality, ethics clearance and contacts. There was also an option for participants to download and keep a copy of the full ‘Participant information sheet’. Participants gave informed consent by ticking in a designated box at the end of the page. Upon completion of the survey, each participant was compensated £2.40 for their time.

### 2.2 Variables and Measurements

#### 2.2.1 Financial Well‐Being

Financial well‐being of participants was assessed through six questions based on the framework of FT proposed by Witte et al. [11]. The questions covered the following subdomains of the framework (i) active financial spending and impact (e.g., debt status, difficulties in paying rent/mortgage); (ii) resort to passive financial resources (e.g., use of savings, selling possessions); (iii) support seeking (e.g., applying for welfare benefits); and (iv) coping lifestyle (e.g., alter usual activities to cope with cancer‐related expenses).

#### 2.2.2 HRQoL (Including EQ‐5D‐5L Descriptive System, EQ‐VAS Score, and Utility Values)

*The EQ‐5D‐5L descriptive system* for HRQoL consists of five dimensions (Mobility, Self‐care, Usual Activities, Pain/Discomfort, Anxiety/Depression). Each dimension has five severity levels from: ‘no problems’, ‘slight problems’, ‘moderate problems’, ‘severe problems’, and ‘unable to/extreme problems’.

##### 2.2.2.1 Perceived Rating of Overall Health Status (EQ‐VAS Score)

Participants rated their overall current HRQoL (called the EQ‐VAS score) by placing a mark on a standard vertical 20 cm visual analogue scale (EQ‐VAS) which ranged from 0 (the worst imaginable health state) to 100 (the best imaginable health state).

##### 2.2.2.2 Utility Values

Norm‐referenced utility values range from 0 (a state as bad as being dead) to 1 (full health state). Negative values represent health states that are considered to be ‘worse than dead’. According to the UK National Institute for Health and Care Excellence (NICE) guidance, as there was currently no acceptable value set for EQ‐5D‐5L, UK utility values should be calculated by mapping the 5 L descriptive system data onto the 3 L value set [18]. We used the mapping function developed by Hernandez Alava et al. [19] and the UK 3L value set by Dolan [20] to calculate UK utility values. US utility values were derived from EQ‐5D‐5L value set by Pickard et al. [21].

#### 2.2.3 Covariates

The main independent variable was FT based on COST score (no FT if COST score ≥ 26, mild FT if COST = 14–25, and moderate/severe FT if COST = 0–13. This is the grading system proposed by the original author of COST [22]). Co‐variates included ‘age’, ‘sex’, ‘education’, ‘marital status’, ‘occupation’, ‘household monthly income’, ‘cancer stage at diagnosis’, ‘time since diagnosis’, and ‘treatment status’ (patient vs. survivor). The treatment status was derived from the question ‘Are you in complete remission or N.E.D.—no evidence of disease state?’ The answers, ‘No, not yet’ or ‘I expect to be on treatment for the rest of my life’ were categorised as indicating ‘patients.’ The answer, ‘Yes’ was used to categorise ‘survivors.’ Previous studies in the UK and US about factors that were associated with HRQoL among cancer patients/survivors and a systematic review of global studies about the topic [23, 24, 25, 26] were used to select covariates and guide analysis. ‘Country’ (UK vs. US) was included as a covariate in the comparative between‐country analysis.

### 2.3 Statistical Analysis

Descriptive statistics (e.g., proportion for categorial variables; mean and standard deviation‐SD for continuous variables) were used to describe sociodemographic and clinical characteristics. Regarding financial well‐being, EQ‐5D‐5L descriptive system, EQ‐VAS scores, and utility values, Chi‐squared (*χ* ^2^) tests, independent *t*‐tests, Mann Whitney, and Kruskal Wallis tests were used to assess the differences between groups with different severity levels of FT.

Tobit regression models were used to analyse EQ‐VAS scores and utility values as this approach is for the censored nature of EQ‐5D data (bounds at full health and worst health state) [27]. The models initially included all covariates based on a literature review and binary analyses. A backward elimination approach and the model goodness‐of‐fit based on Akaike and Bayesian information criteria (AIC and BIC) were used to determine the final models for reporting.

Tests of statistical significance were 2‐sided; the cut‐off for statistical significance was 0.05. All statistical procedures were conducted in STATA 15.0.

## 3 Results

### 3.1 Characteristics of Study Participants

Participants' characteristics are presented in Table 1. The mean age (SD) of participants was 52 (14) years; 53% resided in the UK; 67% were female; 69% were married or in a relationship; 62% were working (either full‐time/parttime or self‐employed); 58% completed at least high school education; 72% were diagnosed with early‐stage cancer (stage 0/I/II); and 91% had time since diagnosis > 1 year.

**TABLE 1:** Sociodemographic and clinical characteristics of the sample.

	Total *n* (%)	Remission/N.E.D. *n* (%)	Patient (undergoing treatment) *n* (%)	*p* *
	600 (100.0)	465 (77.5)	135 (22.5)	
Age, mean (SD)	52 (14)	52 (14)	53 (12)	0.54
Race				
White	528 (88.0)	411 (88.4)	117 (86.7)	0.59
Non‐White	72 (12.0)	54 (11.6)	18 (13.3)	
Country of residence				
United Kingdom	319 (53.2)	247 (53.1)	72 (53.3)	0.96
United States of America	281 (46.8)	218 (46.9)	63 (46.7)	
Sex				
Female	399 (66.9)	307 (66.6)	92 (68.1)	0.74
Male	197 (33.1)	154 (33.4)	43 (31.9)	
Marital status				
Single/separated/divorced/widow	186 (31.1)	144 (31.0)	42 (31.1)	0.99
Married/in relationship	413 (68.9)	320 (69.0)	93 (68.9)	
Highest education level				
Undergraduate and above	142 (23.7)	111 (23.9)	31 (23.0)	0.07
Technical/community college	111 (18.5)	96 (20.6)	15 (11.1)	
High school diploma/A‐levels	221 (36.8)	164 (35.3)	57 (42.2)	
Secondary education or lower	126 (21.0)	94 (20.2)	32 (23.7)	
Occupational status				
Employed full‐time	212 (35.3)	166 (35.7)	46 (34.1)	0.13
Employed part‐time/Self‐employed	158 (26.3)	126 (27.1)	32 (23.7)	
Unemployed	24 (4.0)	20 (4.3)	4 (3.0)	
Other	31 (5.2)	24 (5.2)	7 (5.2)	
Retired	126 (21.0)	99 (21.3)	27 (20.0)	
Disabled/too ill to work	49 (8.2)	30 (6.5)	19 (14.1)	
Household weekly income, before tax				
Up to £200 (up to $250)	45 (7.5)	36 (7.7)	9 (6.7)	1.00
£200–£399 ($250–$499)	85 (14.2)	65 (14.0)	20 (14.8)	
£400–£599 ($500–$749)	114 (19.0)	89 (19.1)	25 (18.5)	
£600–£799 ($750–$999)	89 (14.8)	70 (15.1)	19 (14.1)	
£800–£999 ($1000–$1249)	66 (11.0)	52 (11.2)	14 (10.4)	
£1000–£1199 ($1250–$1499)	48 (8.0)	36 (7.7)	12 (8.9)	
£1200–£1399 ($1500–$1749)	41 (6.8)	32 (6.9)	9 (6.7)	
£1400 or above ($1750 or above)	112 (18.7)	85 (18.3)	27 (20.0)	
Stage of cancer at diagnosis				
Stage 0/I	221 (45.3)	181 (48.4)	40 (35.1)	< 0.001
Stage II	132 (27.0)	93 (24.9)	39 (34.2)	
Stage III	91 (18.6)	76 (20.3)	15 (13.2)	
Stage IV (metastatic)	44 (9.0)	24 (6.4)	20 (17.5)	
Time since diagnosis				
≤ 1 year	56 (9.5)	23 (5.1)	33 (24.4)	< 0.001
> 1 year	533 (90.5)	431 (94.9)	102 (75.6)	

Abbreviations: N.E.D., no evidence of disease; SD, standard deviation.

* Results of Chi‐squared tests compared between participants in the patient group (undergoing treatment) and remission group.

The financial well‐being of participants is presented in Table 2. Within each country, participants who did not experience FT had significantly better financial well‐being. For example, the proportion of UK participants who were in debt due to cancer diagnosis and treatment among those who had FT was 32% compared to 9% among those who had not have FT (odds ratio‐OR = 4.65, 95% CI: 2.44–8.84). Similarly, it was respectively 54% vs. 9% in the US (OR = 12.6, 95% CI: 5.70–27.9). In both countries, 62% of participants with FT had to use their savings to pay expenses related to cancer diagnosis and treatment, significantly higher than 17%–27% among those without FT (UK and US, respectively).

**TABLE 2:** Financial well‐being of US and UK cancer patients/survivors.

	United Kingdom			United States			UK versus US
	No FT *n* (%)	Had FT *n* (%)	*p* *	No FT *n* (%)	Had FT *n* (%)	*p* *	*p* **
*n* (%)	214 (67.1)	105 (32.9)		128 (45.6)	153 (54.4)		
Ever been in debt cancer due to cancer diagnosis/treatment	20 (9.3)	34 (32.4)	< 0.001	11 (8.6)	83 (54.2)	< 0.001	0.001
Amount of debt (I$), median (IQR)		7200 (12960)			11,500 (23000)		0.012
Spend savings to pay for expense related to cancer diagnosis/treatment	37 (17.3)	65 (61.9)	< 0.001	34 (26.6)	94 (61.4)	< 0.001	0.940
Percentage of savings spent on cancer related expenses, mean (SD)	30 (32)	51 (37)	< 0.001	29 (28)	66 (35)	< 0.001	0.012^a^
Difficulties in paying rent/mortgage because of cancer diagnosis/treatment	9 (4.2)	37 (35.2)	< 0.001	4 (3.1)	59 (38.6)	< 0.001	0.587
Sell possessions because of cancer diagnosis/treatment	6 (2.8)	27 (25.7)	< 0.001	3 (2.3)	35 (22.9)	< 0.001	0.600
Alter usual activities to cope with cancer related expenses	49 (22.9)	77 (73.3)	< 0.001	38 (29.7)	109 (71.2)	< 0.001	0.713
Apply for state benefits to help with financial situation	37 (17.3)	61 (58.1)	< 0.001	18 (14.1)	56 (36.6)	< 0.001	0.001

Abbreviations: FT, financial toxicity; I$, International US dollar; IQR, interquartile range; SD, standard deviation.

* Results of Chi‐square tests compared between participants who did not experience financial toxicity and those who did.

** Results of Chi‐square tests compared between participants who had FT in the UK versus US.

^a^ Results of independent *t*‐test compared between participants who had FT in the UK versus US.

Among those with FT in UK & US, nearly 40% faced difficulties in paying rent or mortgage; approximately 25% had to sell possessions; and more than 70% had to alter usual activities to cope with the cost of cancer diagnosis and treatment. These figures did not significantly differ between the two countries.

Difference between UK & US were evident in the proportion of those with FT who were in debt or applied for welfare as well as the amount of debt and percentage of savings exhausted to pay for cancer diagnosis and treatment. Among those with FT, 54% US participants were in debt with median (IQR) of debt at I$11,500 (23,000), compared to 32% in the UK with median (IQR) of debt at I$ 7200 (12,960). The odds of US participants with FT who were in debt were 2.48 times higher than in the UK (OR = 2.48, 95% CI: 1.46–4.21). The amount of debt born by US participants was also significantly higher than that of the UK. In contrast, the proportion of US participants with FT who applied for welfare was significantly lower than in the UK (37% vs. 58%, respectively, OR = 0.42, 95% CI: 0.25–0.70).

Table 3 shows participants' EQ‐5D‐5L health profile across five health dimensions, by country and the severity of FT. In all five dimensions, a significantly higher proportion of participants who had experienced mild or moderate/severe FT reported problems compared to those who had not had FT (Chi‐square tests, all *p* < 0.001). This pattern was the same regardless of participant's residency in the UK or US. Overall, anxiety/depression was the dimension in which the highest proportion of participants reported having any problems. In the UK, 97% of participants with moderate/severe FT and 76% of participants with mild FT reported having anxiety/depression, compared to 53% among those who did not have FT (OR = 33, 95% CI: 4–276 and OR = 2.85, 95% CI: 1.51–5.38, respectively). Similar patterns were observed in the US with 93% and 80% respectively, compared to 50% (OR = 12.4, 95% CI: 4.22–36.5 and OR = 4.06, 95% CI: 2.08–7.91, respectively). In all five dimensions, there was no significant difference between two countries regarding the proportion of those with FT who reported having problems.

**TABLE 3:** Proportion of US and UK cancer patients/survivors who reported problems in each EQ‐5D dimension, by severity of financial toxicity.

	United Kingdom				United States			
	No FT *n* (%)	Mild FT *n* (%)	Moderate/Severe FT *n* (%)	*p* *	No FT *n* (%)	Mild FT *n* (%)	Moderate/Severe FT *n* (%)	*p* *
*n* (%)	214 (67.1)	67 (21.0)	38 (11.9)		128 (45.6)	86 (30.6)	67 (23.8)	
Mobility								
No problems	164 (76.6)	38 (56.7)	12 (31.6)	< 0.001	108 (84.4)	53 (61.6)	30 (44.8)	< 0.001
Slight problems	30 (14.0)	15 (22.4)	10 (26.3)		14 (10.9)	22 (25.6)	18 (26.9)	
Moderate problems	15 (7.0)	10 (14.9)	11 (28.9)		5 (3.9)	10 (11.6)	11 (16.4)	
Severe problems	3 (1.4)	3 (4.5)	4 (10.5)		1 (0.8)	1 (1.2)	7 (10.4)	
Unable to do	2 (0.9)	1 (1.5)	1 (2.6)		0 (0.0)	0 (0.0)	1 (1.5)	
Self‐care								
No problems	200 (93.5)	50 (74.6)	17 (44.7)	< 0.001	123 (96.1)	70 (81.4)	45 (67.2)	< 0.001
Slight problems	12 (5.6)	13 (19.4)	11 (28.9)		4 (3.1)	12 (14.0)	14 (20.9)	
Moderate problems	1 (0.5)	3 (4.5)	7 (18.4)		1 (0.8)	4 (4.7)	8 (11.9)	
Severe problems	1 (0.5)	1 (1.5)	3 (7.9)		0 (0.0)	0 (0.0)	0 (0.0)	
Usual activities								
No problems	149 (69.6)	29 (43.3)	5 (13.2)	< 0.001	106 (82.8)	42 (48.8)	17 (25.4)	< 0.001
Slight problems	50 (23.4)	21 (31.3)	11 (28.9)		16 (12.5)	32 (37.2)	22 (32.8)	
Moderate problems	11 (5.1)	12 (17.9)	12 (31.6)		2 (1.6)	10 (11.6)	18 (26.9)	
Severe problems	4 (1.9)	4 (6.0)	8 (21.1)		4 (3.1)	2 (2.3)	10 (14.9)	
Unable to do	0 (0.0)	1 (1.5)	2 (5.3)		0 (0.0)	0 (0.0)	0 (0.0)	
Pain/discomfort (PD)								
No PD	107 (50.0)	19 (28.4)	6 (15.8)	< 0.001	69 (53.9)	19 (22.1)	10 (14.9)	< 0.001
Slight PD	76 (35.5)	21 (31.3)	8 (21.1)		45 (35.2)	37 (43.0)	20 (29.9)	
Moderate PD	26 (12.1)	23 (34.3)	13 (34.2)		12 (9.4)	19 (22.1)	22 (32.8)	
Severe PD	4 (1.9)	4 (6.0)	10 (26.3)		1 (0.8)	9 (10.5)	13 (19.4)	
Extreme PD	1 (0.5)	0 (0.0)	1 (2.6)		1 (0.8)	2 (2.3)	2 (3.0)	
Anxiety/depression (AD)								
Not AD	101 (47.2)	16 (23.9)	1 (2.6)	< 0.001	64 (50.0)	17 (19.8)	5 (7.5)	< 0.001
Slightly AD	81 (37.9)	29 (43.3)	7 (18.4)		45 (35.2)	31 (36.0)	14 (20.9)	
Moderately AD	23 (10.7)	15 (22.4)	18 (47.4)		15 (11.7)	26 (30.2)	31 (46.3)	
Severely AD	9 (4.2)	5 (7.5)	8 (21.1)		3 (2.3)	10 (11.6)	10 (14.9)	
Extremely AD	0 (0.0)	2 (3.0)	4 (10.5)		1 (0.8)	2 (2.3)	7 (10.4)	

Abbreviations: AD, anxiety/depression; FT, financial toxicity; PD, pain/discomfort.

* Results of Chi‐squared tests compared among groups with different severity of financial toxicity.

**FIGURE 1:**
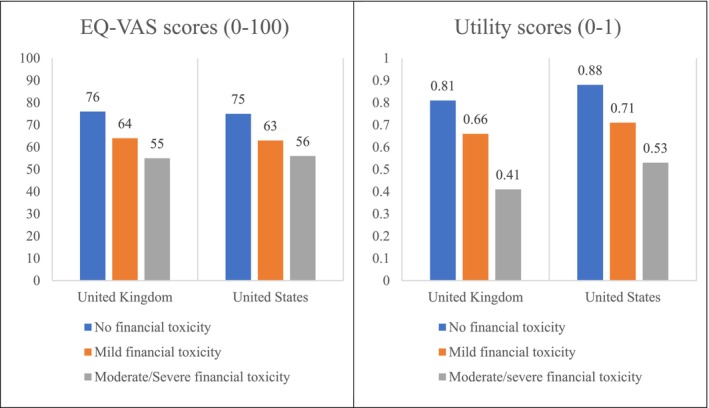
EQ‐VAS/utility scores among US & UK cancer patients/survivors according to severity of financial toxicity.

The correlation coefficient between FT (COST score) and HRQoL (utility values) was 0.53 (95% CI: 0.47–0.59). Figure 1 shows EQ‐VAS/utility scores among US & UK cancer patients/survivors facing different severity of FT. The mean (SD) EQ‐VAS of participants with no FT was 76 (15) in the UK and 75 (17) in the US—significantly higher than participants with mild FT (64 (18) and 63 (18), respectively) and participants with moderate/severe FT (55 (20) and 56 (21), respectively) (Kruskal‐Wallis tests, *p* < 0.001). The mean (SD) utility values of participants with no FT were 0.81 (0.17) in the UK and 0.88 (0.16) in the US which were significantly higher than participants with mild FT (0.66 (0.23) and 0.71 (0.25), respectively) and participants with moderate/severe FT (0.41 (0.30) and 0.53 (0.30), respectively) (Kruskal‐Wallis tests, *p* < 0.001). There were no significant differences at any severity level of FT between the UK and US regarding EQ‐VAS scores. Between‐country statistically significant differences were not observed regarding the utility values of participants except for the group with moderate/severe FT (Mann Whitney test, *p* = 0.0369).

In the multivariable Tobit models, compared with participants who did not experience FT, participants with mild FT reported a 10.75 point lower EQ‐VAS score (*β* = −10.75, 95% CI: −14.45, −7.05) and 0.13 point lower utility values (*β* = 0.13, 95% CI: −0.18, −0.08); while participants with moderate/severe FT reported a 14.65 point lower EQ‐VAS score (*β* = −14.65, 95% CI: −19.54, −9.76) and 0.28 point lower utility values (*β* = −0.28, 95% CI: −0.34, −0.21) (Table 4).

**TABLE 4:** Tobit model analyses of EQ‐5D‐5L utility scores and EQ‐VAS scores.

*n* = 486	Self‐rated health on visual analogue scale (EQ‐VAS score)		Utility score (EQ‐5D‐3L index value)	
	*β* coeff	95% CI	*β* coeff	95% CI
Financial toxicity				
No^a^	0.00	—	0.00	—
Mild	−10.75**	[−14.45, −7.05]	−0.13**	[−0.18, −0.08]
Moderate/Severe	−14.65**	[−19.54, −9.76]	−0.28**	[−0.34, −0.21]
Age	0.01	[−0.12, 0.13]	0.001	[−0.001, 0.002]
Country of residence				
United Kingdom^a^	0.00	—	0.00	—
United States of America	−1.83	[−4.87, 1.21]	0.09**	[0.05, 0.13]
Sex				
Female^a^	0.00	—	0.00	—
Male	0.02	[−3.20, 3.25]	0.068*	[0.03, 0.10]
Occupation				
Employed full‐time^a^	0.00	—	0.00	—
Employed part‐time/Self‐employed	−1.23	[−5.08, 2.62]	−0.01	[−0.06, 0.03]
Unemployed	0.02	[−7.16, 7.19]	−0.02	[−0.10, 0.05]
Other	1.90	[−5.67, 9.48]	0.02	[−0.05, 0.10]
Retired	−4.30	[−9.06, 0.45]	−0.02	[−0.08, 0.03]
Disabled/too ill to work	−14.87**	[−20.97, −8.77]	−0.32**	[−0.43, −0.20]
Stage of cancer at diagnosis^a^				
Stage 0/I	0.00	—	0.00	—
Stage II	−0.71	[−4.32, 2.90]	−0.07*	[−0.12, −0.31]
Stage III	−0.31	[−4.43, 3.80]	−0.07*	[−0.12, −0.21]
Stage IV (metastatic)	−0.93	[−6.71, 4.85]	−0.09*	[−0.17, 0.01]
Treatment status				
Remission/N.E.D.^a^	0.00	—	0.00	—
Patient	0.57	[−3.69, 4.84]	0.03	[−0.01, 0.08]
Lifetime patient	−3.60	[−9.96, 2.76]	−0.02	[−0.09, 0.06]

Abbreviations: CI, confidence interval; N.E.D., no evidence of disease state.

* *p* < 0.05 versus reference group.

** *p* < 0.001 versus reference group.

^a^ Reference group.

Sociodemographic characteristics significantly associated with utility values were country of residence, sex, occupation, and stage of cancer at diagnosis. Specifically, residing in the US and being male were associated with higher utility scores (*β* = 0.09, 95% CI: 0.05,0.13 and *β* = 0.06, 95% CI: 0.03,0.10). Being disabled/too ill to work or being diagnosed at stage II/III/IV of cancer were significantly associated with a negative impact on utility values (*β* = −0.32, 95% CI: −0.43, −0.20 and *β* = −0.07/−0.07/−0.09, respectively, *p* < 0.05).

## 4 Discussion

### 4.1 Financial Well‐Being of Cancer Patients and Survivors

Regardless of being a resident in the UK, where treatment is free at point of use, or in the US, where funding arrangements typically see individuals assume a greater degree of personal responsibility for their healthcare costs, a significant percentage of cancer patients and survivors experienced FT. Those who experienced FT had lower financial well‐being compared to participants without FT. Participants with FT compared to participants without FT struggled in all subdomains of financial well‐being that were investigated in this study including being in debt, spending savings, facing difficulties in paying rent or mortgage, selling possessions, altering usual activities to cope with cancer related expenses, and applying for welfare support. Overall, these results concur with the findings from previous research in the US [13, 14] UK [15, 16] Ireland [28], Canada [29], China [30], and Vietnam [3].

Cancer survivors from the UK and US experienced the same problems, but the magnitude of some problems varied. For example, those in the US were more likely to exhaust a greater percentage of their savings and risk being in debt, compared to their UK counterparts. This observation, perhaps, was due to the greater protection afforded by the publicly funded healthcare system in the UK as well as the welfare system. UK participants with FT were 1.5 times more likely to apply for welfare payments compared to US participants. This pattern of results may be due to several factors such as the availability of state benefits, ease of the application process, awareness of benefits available, role of the voluntary (non‐government organisations) sector in signposting and supporting applications or attitudes to receipt of welfare support. For example, an UK study of 1174 cancer patients reported that 96% of benefit claims were successful and contributed £70.30 per week to a patients' income [31]. By contrast in a study of US oncology cancer service ‘navigators’, only 45% indicated that their patients were able to access financial assistance [32].

### 4.2 Health Profile Across Five Health Dimensions

Across the five health dimensions (mobility, self‐care, usual activities, pain/discomfort, and anxiety/depression), regardless of the country of residence, cancer patients and survivors with FT faced more problems compared to participants who did not experience FT. The most affected dimension was anxiety/depression wherein almost every participant with severe/moderate FT reported problems (93%–97%), compared to those without FT (50%–53%). The odds of having anxiety/depression‐related problems, compared to participants without FT, were 3–4 times higher among participants with mild FT and 12–33 times higher among participants with moderate/severe FT. This result is similar to the findings of a systematic review that reported a positive association between FT and anxiety and depression and a 3‐fold increased risk of depression and anxiety in cancer survivors experiencing FT [6].

### 4.3 Health‐Related Quality of Life (Utility Values)

The more severe the level of FT that was experienced by UK and US participants, the lower was their self‐reported HRQoL. This finding is consistent with previous studies and systematic reviews [3, 4, 5, 11, 33, 34, 35, 36]. The result is statistically significant and clinically meaningful. The difference in mean utility values for participants with mild FT or moderate/severe FT compared to participants without FT was 0.13 and 0.28 points, respectively. These values were higher than the minimally important difference (MID) in HRQoL scores based on the US tariffs of 0.078 [37]. MID indicates the amount of change in HRQoL measure that is sufficient to justify treatment change or an intervention's effectiveness [37].

The correlation coefficient between the FT (COST score) and HRQoL (utility values) was 0.53 (95% CI: 0.47–0.59) similar to a recent meta‐analysis which reported a pooled correlation coefficient of 0.49 (95% CI: 0.44–0.54) [36] (data was pooled from only studies that used the COST scale to measure FT). Sociodemographic characteristics that were significantly associated with HRQoL included being male (positive impact), disabled/too ill to work (negative impact), and later stage of cancer at diagnosis (negative impact). These findings are in keeping with the results from previous studies [3, 4, 5, 11, 33, 34, 35, 36].

It is, perhaps, surprising that the Tobit models suggested that being in the US was positively associated with HRQoL given that the study findings showed that being in the US was negatively associated with cancer patients/survivors' financial well‐being, and that FT is more severe in the US compared to the UK (manuscript under review). A possible explanation might be related to societal norms. For example, it might be the case that FT is expected more readily by US participants compared to the UK; and US cancer patients/survivors may not be as affected as their counterparts in the UK where may be FT underestimated and its effects unappreciated as a problem. It may be the case that the UK public healthcare system with ‘free’ treatment leads participants to expect or believe that cancer care cost is not a problem and to be less aware of the burden that comes from non‐medical direct costs (e.g., transportation, special foods, heating) and indirect costs (e.g., loss of income) related to cancer.

### 4.4 Strengths and Limitations

This is the first comparative study to investigate the impact of FT on the HRQoL and financial well‐being of cancer patients and cancer survivors in the UK and US. Furthermore, the use of the standardised EQ‐5D‐5L and COST instruments enabled international comparisons with other studies. The study provides novel and valuable insights into the financial well‐being and HRQoL of cancer patients/survivors, especially regarding the different experiences that were encountered by participants who did or did not report FT as well as UK‐ and US‐resident participants.

It is important to be mindful of study limitations. Firstly, the study used convenience sampling which could lead to the unrepresentativeness of the sample. In addition, it is important to note that our sample was dominated by participants who were White, and the majority were female. Further studies with representative samples are necessary, particularly given the ethnically diverse populations of the UK and US. There is a need to investigate the impact of FT on financial well‐being and HRQoL among non‐White groups. Secondly, this study employed a cross‐sectional survey study design and, so, the causal relationship between FT and HRQoL cannot be inferred. Other study designs such as a longitudinal study are required to fully investigate the nature and direction of relationships. Thirdly, due to unavailability of data, we could not investigate how the types of treatment received were associated with FT and HRQoL. Whether, for example, those in receipt of more expensive therapies or those requiring greater rehabilitation had different experiences to those who had not. Lastly, there was no available acceptable 5L value set for the UK with respect to the EQ‐5D‐5L instrument and we had to map the values across to 3L to derive the utility values for UK participants. Although the mapping function is recommended, it holds some drawbacks when it is applied to a 3L set compared to the 5L values set.

## 5 Conclusion

Cancer patients and survivors with mild or moderate/severe FT showed significantly lower HRQoL in all five health dimensions compared to those who did not experience FT. The biggest impact was with respect to the dimension of anxiety/depression (and the impact is bigger in UK compare to in US) which may be interpreted as suggesting that more research and interventions may be needed to support those with a cancer diagnosis or who are in a post‐treatment phase and in a domain that is often neglected in relation to FT in terms of the ‘psychological response’ (towards increased cancer‐related expenses). Being a participant who was resident in the US (positive impact) and male (positive impact) were associated with higher HRQoL; while being disabled/too ill to work and later stage of cancer at diagnosis were associated with lower HRQoL. Overall, these results point to an urgent need to improve understanding about FT in health systems globally and to efforts designed to mitigate FT in cancer treatment and recovery.

## Author Contributions

**Tran Thu Ngan:** conceptualization (equal), data curation (lead), formal analysis (lead), funding acquisition (lead), investigation (lead), methodology (equal), writing – original draft (lead), writing – review and editing (equal). **Emily Tonorezos:** conceptualization (equal), writing – review and editing (equal). **Michael Donnelly:** conceptualization (equal), writing – review and editing (equal). **Ciaran O'Neill:** conceptualization (equal), formal analysis (supporting), writing – review and editing (equal).

## Ethics Statement

The study was carried out in accordance with the ethical standards of the Helsinki Declaration. It received the ethical approval No. MHLS 22_178 dated 18 January 2023 from Faculty of Medicine, Health and Life Sciences Research Ethics Committee, Queen's University Belfast, United Kingdom.

## Consent

Informed consent was obtained from all individual participants included in the study. Participants were compensated £2.40 for their time upon completion of the survey.

## Conflicts of Interest

The authors declare no conflicts of interest.

## Précis

Financial toxicity (FT) is associated with poorer financial well‐being and health‐related quality of life (HRQoL) among cancer patients/survivors in the US and UK. The impact of FT on financial well‐being is larger in the US while the impact on HRQoL is worse in the UK.

## Data Availability

The dataset underlying this article cannot be shared due to the restrictions in participant's informed consent ‘All information that you provide will remain confidential and will be accessible only to the researchers conducting this study. Data collected during the study may be published in academic journals and presented at conferences. Such data will be anonymised and presented in aggregated and/or summarised form.’ All aggregated and/or summarised data were presented in this paper.

## References

[1] S. Y. Zafar , J. M. Peppercorn , D. Schrag , et al., “The Financial Toxicity of Cancer Treatment: A Pilot Study Assessing Out‐Of‐Pocket Expenses and the Insured Cancer Patient's Experience,” Oncologist 18, no. 4 (2013): 381–390, 10.1634/theoncologist.2012-0279. 23442307 PMC3639525

[2] S. Y. Zafar and A. P. Abernethy , “Financial Toxicity, Part I: A New Name for a Growing Problem,” Oncology (Williston Park, N.Y.) 27, no. 2 (2013): 80–149. 23530397 PMC4523887

[3] T. T. Ngan , H. Van Minh , M. Donnelly , and C. O'Neill , “Financial Toxicity due to Breast Cancer Treatment in Low‐ and Middle‐Income Countries: Evidence From Vietnam,” Supportive Care in Cancer 29, no. 11 (2021): 6325–6333, 10.1007/s00520-021-06210-z. 33860362 PMC8464564

[4] F. Perrone , C. Jommi , M. Di Maio , et al., “The Association of Financial Difficulties With Clinical Outcomes in Cancer Patients: Secondary Analysis of 16 Academic Prospective Clinical Trials Conducted in Italy,” Annals of Oncology 27, no. 12 (2016): 2224–2229, 10.1093/annonc/mdw433. 27789469

[5] S. N. Rogers , C. N. Harvey‐Woodworth , J. Hare , P. Leong , and D. Lowe , “Patients' Perception of the Financial Impact of Head and Neck Cancer and the Relationship to Health Related Quality of Life,” British Journal of Oral & Maxillofacial Surgery 50, no. 5 (2012): 410–416, 10.1016/j.bjoms.2011.07.026. 22000023

[6] R. J. Chan , L. G. Gordon , C. J. Tan , et al., “Relationships Between Financial Toxicity and Symptom Burden in Cancer Survivors: A Systematic Review,” Journal of Pain and Symptom Management 57, no. 3 (2019): 646, 10.1016/j.jpainsymman.2018.12.003. 30550833

[7] G. L. Smith , M. A. Lopez‐Olivo , P. G. Advani , et al., “Financial Burdens of Cancer Treatment: A Systematic Review of Risk Factors and Outcomes,” Journal of the National Comprehensive Cancer Network 17, no. 10 (2019): 1184–1192, 10.6004/jnccn.2019.7305. 31590147 PMC7370695

[8] K. Honda , B. Gyawali , M. Ando , et al., “Prospective Survey of Financial Toxicity Measured by the Comprehensive Score for Financial Toxicity in Japanese Patients With Cancer,” Journal of Global Oncology 5 (2019): 1–8, 10.1200/JGO.19.00003. PMC6550026 31070981

[9] A. Fabian , J. Domschikowski , W. Greiner , et al., “Financial Toxicity in Cancer Patients Treated With Radiotherapy in Germany—A Cross‐Sectional Study,” Strahlentherapie Und Onkologie: Organ der Deutschen Rontgengesellschaft 198 (2022): 1053–1061, 10.1007/s00066-022-01936-z. PMC9700565 35467099

[10] M. I. Fitch and C. J. Longo , “Emerging Understanding About the Impact of Financial Toxicity Related to Cancer: Canadian Perspectives,” Seminars in Oncology Nursing 37, no. 4 (2021): 151174, 10.1016/j.soncn.2021.151174. 34266710

[11] J. Witte , K. Mehlis , B. Surmann , et al., “Methods for Measuring Financial Toxicity After Cancer Diagnosis and Treatment: A Systematic Review and Its Implications,” Annals of Oncology 30, no. 7 (2019): 1061–1070, 10.1093/annonc/mdz140. 31046080 PMC6637374

[12] T. T. Ngan , T. H. Tien , M. Donnelly , and C. O'Neill , “Financial Toxicity Among Cancer Patients, Survivors and Their Families in the United Kingdom: A Scoping Review,” Journal of Public Health 45, no. 4 (2023): e702–e713, 10.1093/pubmed/fdad143. 37541834 PMC10687873

[13] N. Iragorri , C. de Oliveira , N. Fitzgerald , and B. Essue , “The Out‐Of‐Pocket Cost Burden of Cancer Care—A Systematic Literature Review,” Current Oncology 28, no. 2 (2021): 1216–1248, 10.3390/curroncol28020117. 33804288 PMC8025828

[14] A. M. Gilligan , D. S. Alberts , D. J. Roe , and G. H. Skrepnek , “Death or Debt? National Estimates of Financial Toxicity in Persons With Newly‐Diagnosed Cancer,” American Journal of Medicine 131, no. 10 (2018): 1187–1199, 10.1016/j.amjmed.2018.05.020. 29906429

[15] Macmillan Cancer Support , Cancer's Hidden Price Tag: Revealing the Costs Behind the Illness (London: Macmillan Cancer Support, 2013).

[16] Macmillan Cancer Support , “No Small Change: Time to Act on the Financial Impact of Cancer,” 2017.

[17] T. T. Ngan , E. Tonorezos , M. Donnelly , and C. O'Neill , “Financial Toxicity Among Cancer Patients and Survivors: A Comparative Study of the United Kingdom and United States,” Preprint at Research Square (2024): v1, 10.21203/rs.3.rs-4613344/v1. PMC11733585 39811923

[18] National Institute for Health and Care Excellence (NICE) , “Position Statement on use of the EQ‐5D‐5L Value set for England [Technology Appraisal Guidance]. London: National Institute for Health and Care Excellence (NICE),” 2019, https://www.nice.org.uk/about/what‐we‐do/our‐programmes/nice‐guidance/technology‐appraisal‐guidance/eq‐5d‐5l.

[19] M. Hernández Alava , S. Pudney , and A. Wailoo , “Estimating the Relationship Between EQ‐5D‐5L and EQ‐5D‐3L: Results From a UK Population Study,” PharmacoEconomics 41, no. 2 (2023): 199–207, 10.1007/s40273-022-01218-7. 36449173 PMC9883358

[20] P. Dolan , “Modeling Valuations for EuroQol Health States,” Medical Care 35, no. 11 (1997): 1095–1108. 9366889 10.1097/00005650-199711000-00002

[21] A. S. Pickard , E. H. Law , R. Jiang , et al., “United States Valuation of EQ‐5D‐5L Health States Using an International Protocol,” Value in Health 22, no. 8 (2019): 931–941, 10.1016/j.jval.2019.02.009. 31426935

[22] J. A. De Souza , K. Wroblewski , E. Proussaloglou , L. Nicholson , A. Hantel , and Y. Wang , “Validation of a Financial Toxicity (FT) Grading System,” Journal of Clinical Oncology 35, no. 15_suppl (2017): 6615, 10.1200/JCO.2017.35.15_suppl.6615.

[23] B. Akbar Javan , R. Samira , R. Sima , et al., “Global Quality of Life in Breast Cancer: Systematic Review and Meta‐Analysis,” BMJ Supportive &Amp;Amp; Palliative Care 13, no. e3 (2023): e528, 10.1136/bmjspcare-2022-003642. PMC10850719 35710706

[24] P. Hopwood , J. Haviland , J. Mills , G. Sumo , and M. Bliss , “The Impact of Age and Clinical Factors on Quality of Life in Early Breast Cancer: An Analysis of 2208 Women Recruited to the UK START Trial (Standardisation of Breast Radiotherapy Trial),” Breast 16, no. 3 (2007): 241–251, 10.1016/j.breast.2006.11.003. 17236771

[25] S. Manne , K. Devine , S. Hudson , et al., “Factors Associated With Health‐Related Quality of Life in a Cohort of Cancer Survivors in New Jersey,” BMC Cancer 23, no. 1 (2023): 664, 10.1186/s12885-023-11098-5. 37452275 PMC10349446

[26] H. Carreira , R. Williams , H. Dempsey , S. Stanway , L. Smeeth , and K. Bhaskaran , “Quality of Life and Mental Health in Breast Cancer Survivors Compared With Non‐cancer Controls: A Study of Patient‐Reported Outcomes in the United Kingdom,” Journal of Cancer Survivorship 15, no. 4 (2021): 564–575, 10.1007/s11764-020-00950-3. 33089480 PMC8272697

[27] N. Devlin , D. Parkin , and B. Janssen , Methods for Analysing and Reporting EQ‐5D Data (Cham: Springer, 2020). 33347096

[28] L. Sharp and A. Timmons , The Financial Impact of a Cancer Diagnosis (Cork, Ireland: National Cancer Registry Ireland, 2010), https://www.ncri.ie/sites/ncri/files/pubs/FinancialImpactofaCancerDiagnosis%28FullReport%29.pdf.

[29] K. N. Mahon , S. N. Garland , G. Eaton , et al., “The Financial Impact of Cancer on Canadian Young Adults,” Journal of Cancer Survivorship 17, no. 1 (2023): 174–186, 10.1007/s11764-021-00998-9. 33586129

[30] M. Su , J. Lao , N. Zhang , et al., “Financial Hardship in Chinese Cancer Survivors,” Cancer 126, no. 14 (2020): 3312–3321, 10.1002/cncr.32943. 32396242

[31] S. Moffatt , E. Noble , and M. White , “Addressing the Financial Consequences of Cancer: Qualitative Evaluation of a Welfare Rights Advice Service,” PLoS One 7, no. 8 (2012): e42979, 10.1371/journal.pone.0042979. 22900073 PMC3416776

[32] J. C. Spencer , C. A. Samuel , D. L. Rosenstein , et al., “Oncology Navigators' Perceptions of Cancer‐Related Financial Burden and Financial Assistance Resources,” Supportive Care in Cancer 26, no. 4 (2018): 1315–1321, 10.1007/s00520-017-3958-3. 29124417

[33] H. R. Abrams , S. Durbin , C. X. Huang , et al., “Financial Toxicity in Cancer Care: Origins, Impact, and Solutions,” Translational Behavioral Medicine 11, no. 11 (2021): 2043–2054, 10.1093/tbm/ibab091. 34850932

[34] C. Barbaret , C. Brosse , W. Rhondali , et al., “Financial Distress in Patients With Advanced Cancer,” PLoS One 12, no. 5 (2017): e0176470, 10.1371/journal.pone.0176470. 28545063 PMC5436643

[35] S. Pauge , B. Surmann , K. Mehlis , et al., “Patient‐Reported Financial Distress in Cancer: A Systematic Review of Risk Factors in Universal Healthcare Systems,” Cancers 13, no. 19 (2021), 10.3390/cancers13195015. PMC8508394 34638499

[36] S. Pangestu and F. Rencz , “Comprehensive Score for Financial Toxicity and Health‐Related Quality of Life in Patients With Cancer and Survivors: A Systematic Review and Meta‐Analysis,” Value in Health 26, no. 2 (2023): 300–316, 10.1016/j.jval.2022.07.017. 36064514

[37] E. B. Henry , L. E. Barry , A. P. Hobbins , N. S. McClure , and C. O'Neill , “Estimation of an Instrument‐Defined Minimally Important Difference in EQ‐5D‐5L Index Scores Based on Scoring Algorithms Derived Using the EQ‐VT Version 2 Valuation Protocols,” Value in Health 23, no. 7 (2020): 936–944, 10.1016/j.jval.2020.03.003. 32762996

